# FAMILIAL VISITATION OF LONG TERM CARE FACILITY RESIDENTS: DIFFERENTIATING SOCIAL BEHAVIORS FROM CARE COORDINATION

**DOI:** 10.1093/geroni/igae098.4187

**Published:** 2024-12-31

**Authors:** Caroline Collins-Pisano, Rachel Weiskittle

**Affiliations:** University of Colorado, Colorado Springs, Colorado Springs, Colorado, United States; University of Colorado, Colorado Springs, Colorado Springs, Colorado, United States

## Abstract

High rates of loneliness persist among residents of long-term care (LTC) facilities despite frequent familial visitation. Most studies on LTC visitation do not differentiate social behavior (e.g., talking to the resident about their interests) from care coordination (e.g., attending the resident’s care conferences). It is possible but unknown if these distinct forms of visitation impact resident loneliness differently. Past research has identified barriers to overall LTC facility visitation, such as family member demographics (e.g., gender, income) and relational factors (e.g., closeness, history of care provision). The aim of the present study was to determine whether these demographic and relational factors related to social behavior and care coordination differently. Community-dwelling adults aged 18 and older who endorsed having a close friend or relative currently residing in a LTC facility (N = 175) recruited via ResearchMatch completed an online survey regarding their demographics, LTC visiting behaviors, and relationship with the resident. Overall, results indicated demographic variables were not significantly associated with either social behavior or care coordination, but relational factors were significant predictors of both outcomes. Individuals who reported higher closeness with the resident and who had been their primary caregiver demonstrated significantly greater care coordination involvement and social behavior involvement. Immediate family members were more likely to be involved in care coordination than friends or extended family, but they were not more likely to socially interact with the resident. These findings imply there are distinct facilitators and barriers for care coordination and social involvement that warrant further investigation.

